# Supplementary material for: Mathematical modeling of plant cell fate transitions controlled by hormonal signals
Source: PLoS Comput Biol. 2020 Jul 20;16(7):e1007523. doi: 10.1371/journal.pcbi.1007523 (PMC7392350; doi:10.1371/journal.pcbi.1007523)
Supplement: S1 Text — (PDF) [file pcbi.1007523.s001.pdf]

Supplementary Information S1 Text: Mathematical modeling of plant cell fate transitions controlled by hormonal signals

Filip Z. Klawe[1,⋆], Thomas Stiehl[1,2,3,⋆], Peter Bastian[2], Christophe Gaillochet[4], Jan U. Lohmann[5], and Anna Marciniak-Czochra[1,2,3]

[1] *Institute of Applied Mathematics, Heidelberg University, Heidelberg, Germany*
[2] *Interdisciplinary Center for Scientific Computing, Heidelberg University, Heidelberg, Germany*
[3] *Bioquant Center, Heidelberg University, Heidelberg, Germany*
[4] *VIB-UGent Center for Plant Systems Biology, Ghent University, Ghent, Belgium*
[5] *Department of Stem Cell Biology, Centre for Organismal Studies, Heidelberg University, Heidelberg, Germany*
[⋆] *These authors contributed equally*

Functions f and g. The shapes of the functions f and g are depicted in Fig. A.

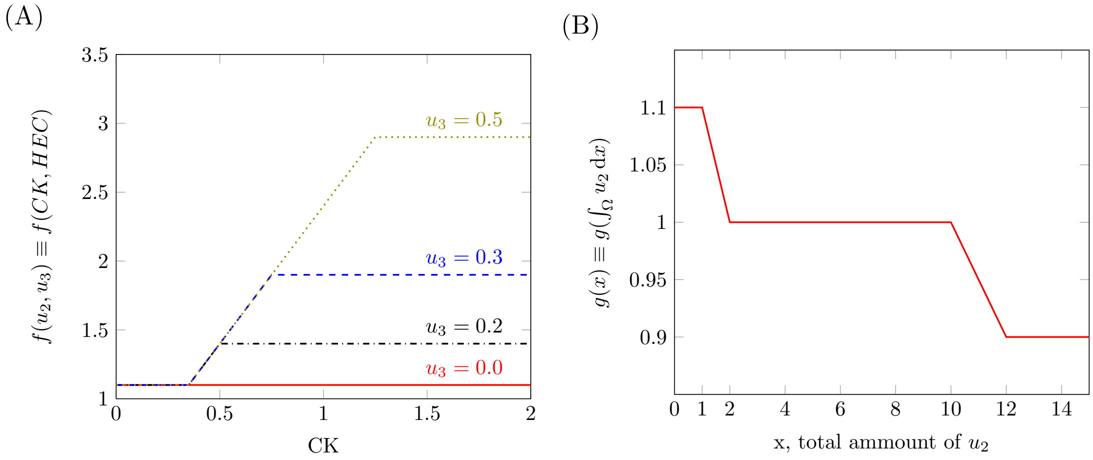

Figure A: **Functions f and g.** (A) The shape of the function f for different concentrations of CK and HEC. (B) The shape of the function g.
