# Supplementary material for: Mathematical modeling of plant cell fate transitions controlled by hormonal signals
Source: PLoS Comput Biol. 2020 Jul 20;16(7):e1007523. doi: 10.1371/journal.pcbi.1007523 (PMC7392350; doi:10.1371/journal.pcbi.1007523)
Supplement: S2 Text — (PDF) [file pcbi.1007523.s002.pdf]

Supplementary Information S2 Text: Mathematical modeling of plant cell fate transitions controlled by hormonal signals

Filip Z. Klawe[1,⋆], Thomas Stiehl[1,2,3,⋆], Peter Bastian[2], Christophe Gaillochet[4], Jan U. Lohmann[5], and Anna Marciniak-Czochra[1,2,3]

[1]*Institute of Applied Mathematics, Heidelberg University, Heidelberg, Germany*
[2]*Interdisciplinary Center for Scientific Computing, Heidelberg University, Heidelberg, Germany*
[3]*Bioquant Center, Heidelberg University, Heidelberg, Germany*
[4]*VIB-UGent Center for Plant Systems Biology, Ghent University, Ghent, Belgium*
[5]*Department of Stem Cell Biology, Centre for Organismal Studies, Heidelberg University, Heidelberg, Germany*
[⋆]*These authors contributed equally*

Additional simulations results.

Different rates of WUS over-expression. We run simulations for different rates of WUS over-expression. In the model this corresponds to different values of c. Depending on the rate of WUS over-expression different dynamics can be observed.

- Mild WUS over-expression ($c = 1.0$) in the whole meristem, Fig. A (A): Inhibition of HEC leads to increased OC differentiation. The OC shrinks and therefore also the WUS and CLV3 concentration in the center of the meristem. The over-expression is too mild to trigger a CLV3 production comparable to that in the center of the wild type CZ. The system approaches a state with decreased WUS and CLV3 concentrations. The OC cell number is different from zero and the state is nonconstant in space.

- Intermediate WUS over-expression ($c = 2.9$) in the whole meristem, Fig. A (B): Inhibition of HEC leads to increased OC differentiation and extinction of the OC cells. Therefore, WUS production in the OC ceases. The system converges to a state that is constant in space and that is maintained by the ubiquitous constant WUS expression. This level of WUS expression, however, leads to CLV3 concentrations lower than the CLV3 concentrations in the center of the wild-type meristem.

- Strong WUS over-expression ($c = 3.1$) in the whole meristem, Fig. A (C): Inhibition of HEC leads to increased OC differentiation and extinction of OC cells. The system converges to a state that is constant in space and that is maintained by the ubiquitous constant WUS expression. In this case the WUS expression is high enough to trigger CLV3 concentrations similar to those in the center of the wild-type meristem. This case reflects what is seen in WUS over-expression experiments [2].

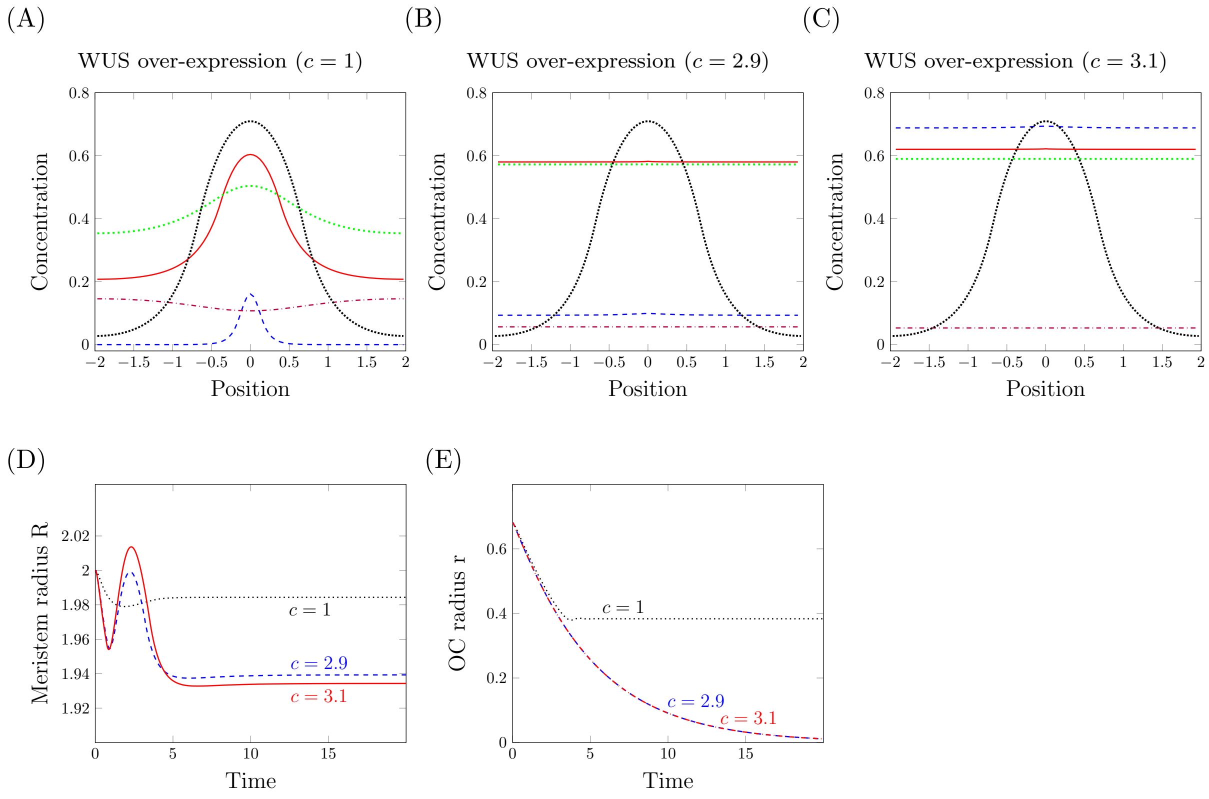

Figure A: **Mild, intermediate and strong WUS over-expression.** Concentrations of signals along the diameter of the meristem, position 0 corresponds to the center: WUS (solid red), CLV3 (dashed blue), CK (dotted green) and HEC (dashdotted purple). WUS over-expression is simulated by adding a constant production c in the whole meristem. (A) Mild ubiquitous WUS over-expression. (B) Intermediate ubiquitous WUS over-expression. (C) Strong ubiquitous WUS over-expression. The equilibrium concentration of WUS in the wild type is shown for comparison (densely dotted black). (D, E) Time evolution of meristem and organizing center (OC) radius.

WUS loss of function. Fig. B shows time evolution of meristem and OC radius.

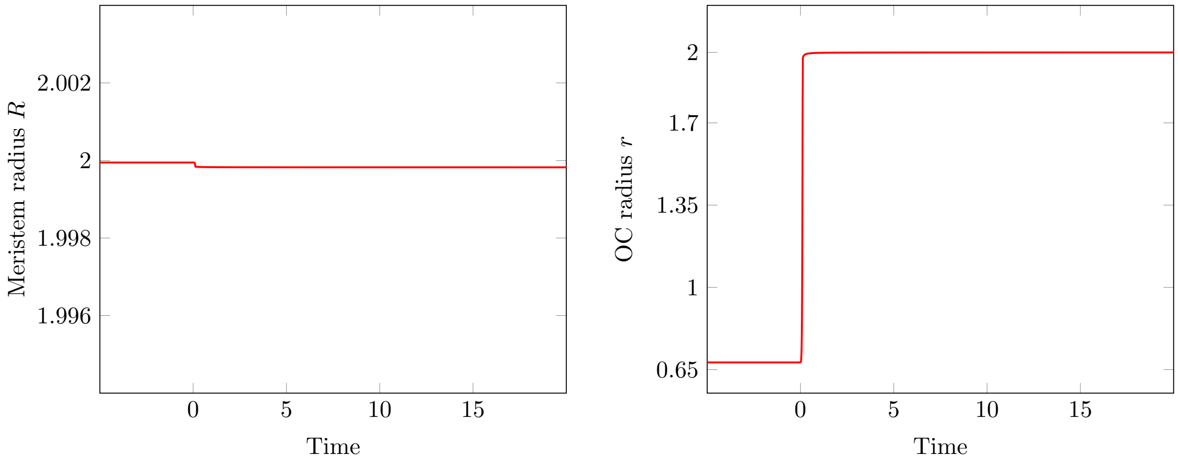

Figure B: **WUS loss of function.** Time evolution of meristem and OC radius. WUS loss of function starts at time $t = 0$. The initial condition is the unperturbed equilibrium of the wild-type meristem.

CLV3 over-expression. Fig. C shows simulations for different levels of CLV3 over-expression. We observe that WUS concentrations are reduced compared to the wild type meristem. Different levels of over-expression lead to qualitatively similar results.

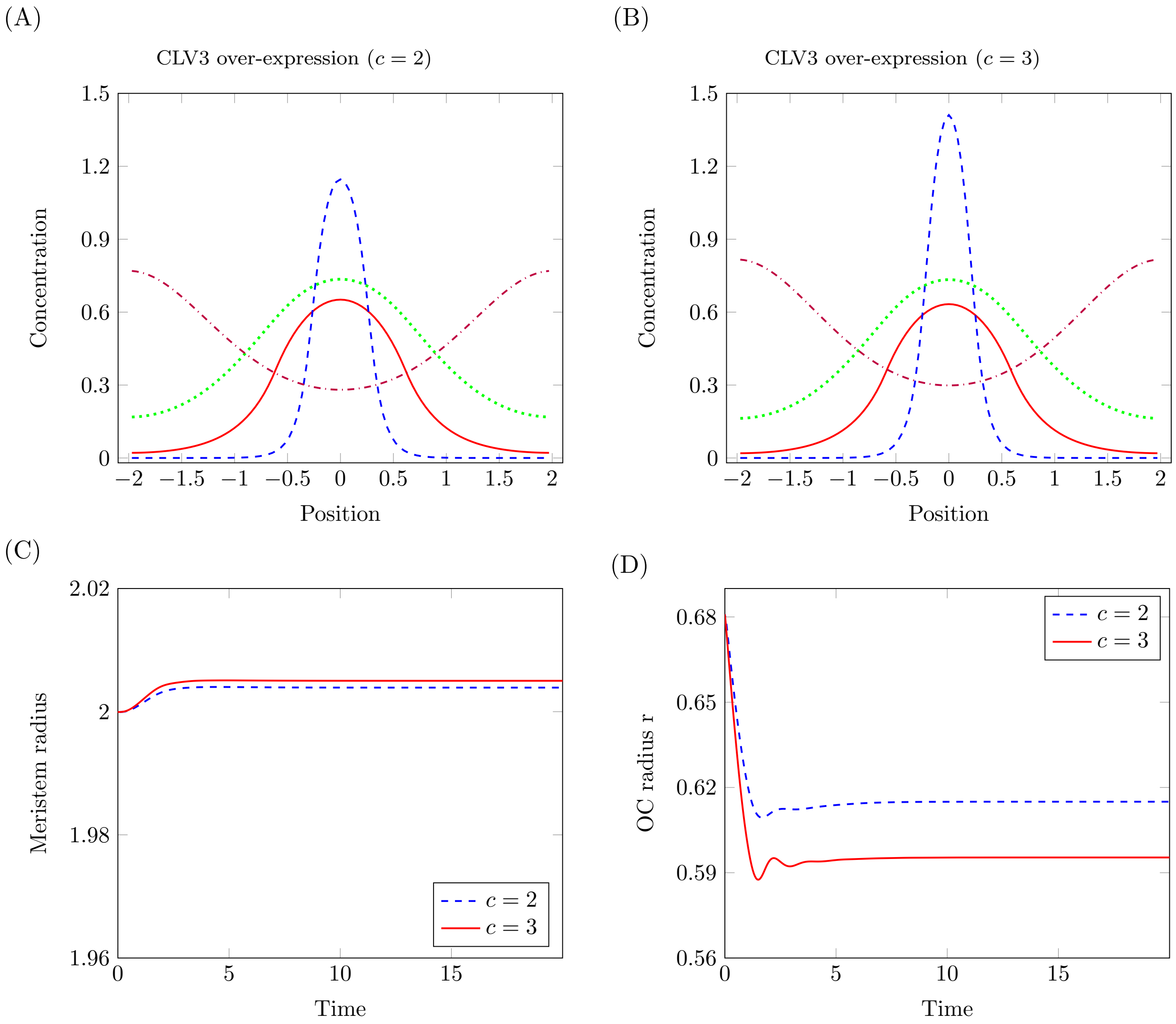

Figure C: **Different levels of CLV3 over-expression.** Concentrations of WUS (red solid), CLV3 (dashed blue), CK (dotted green) and HEC (dashdotted purple) along the diameter of the meristem, position 0 corresponds to the center. CLV3 over-expression takes place in the cells which naturally express CLV3. It is simulated by multiplying the CLV3 production term by a constant c. (A) $c = 2$, (B) $c = 3$, (C-D) time evolution of meristem and OC radius after onset of over-expression.

CLV3 loss of function. Fig. D shows time evolution of meristem and OC radius. CLV3 loss of function leads to an increase of the OC radius.

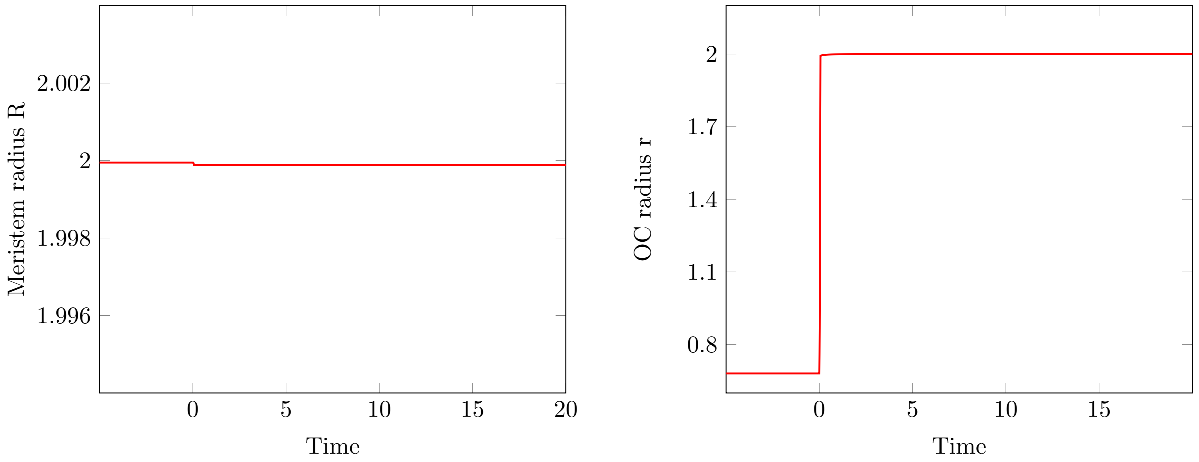

Figure D: **Meristem size in CLV3 loss-of-function.** Time evolution of meristem and OC radius after CLV3 loss of function.

Reduced CK degradation. Fig. E shows simulations for different levels of reduction of CK degradation. Reduced CK degradation is modeled by the following modification of the equation for u_2:

$$\partial_t u_2 = 0.2\Delta u_2 + \left(1 + \frac{4u_3}{1+0.1u_3}\right)\frac{1.4u_0}{1+u_0} - (1.1-c)u_2. \tag{S1}$$

Reduced CK degradation leads to an increase of the OC radius. If the reduction is strong enough, we also observe an increase in meristem radius.

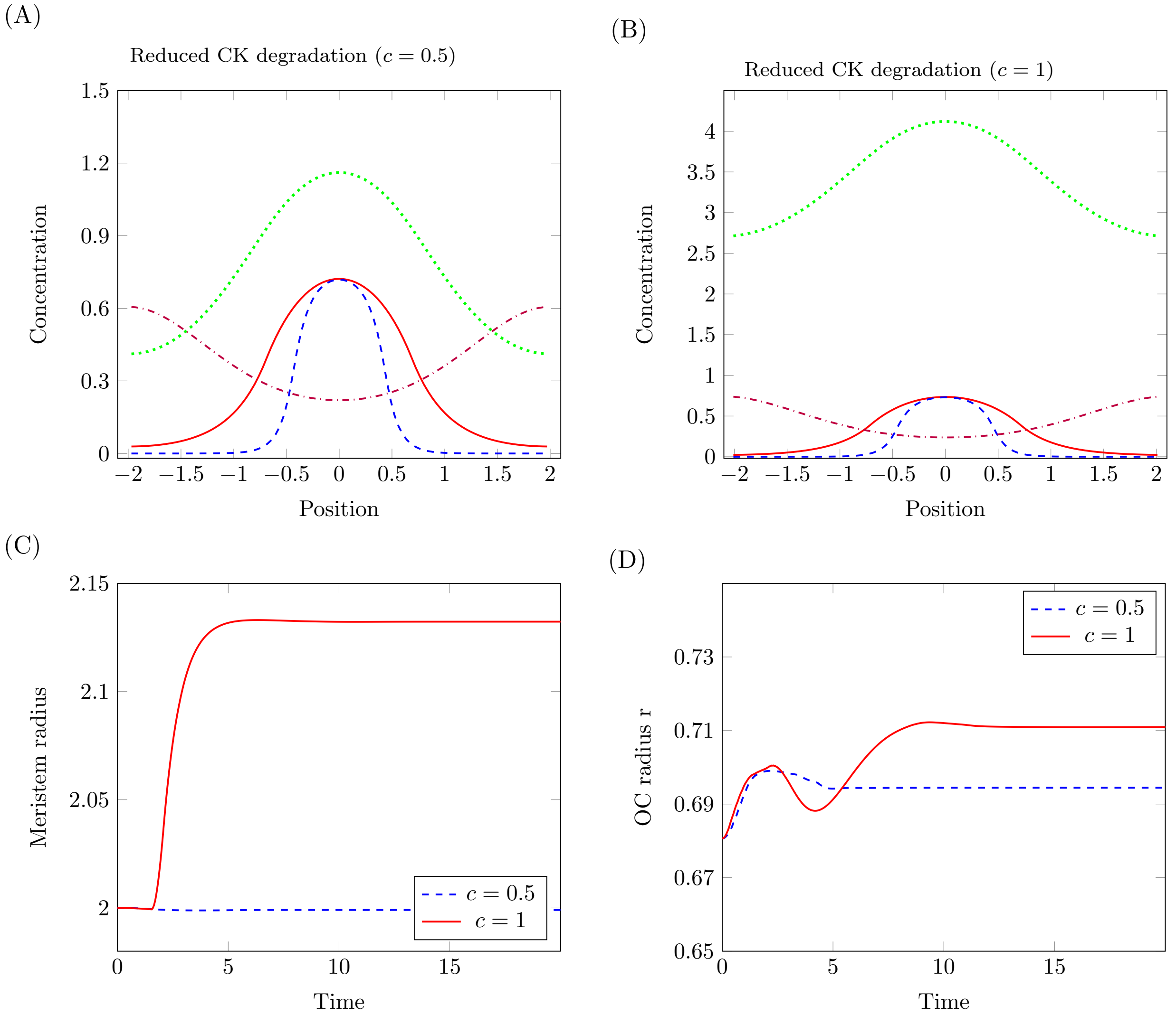

Figure E: **CK over-expression.** Concentrations of WUS (solid red), CLV3 (dashed blue), CK (dotted green) and HEC (dashdotted purple) along the diameter of the meristem, position 0 corresponds to the center. CK over-expression takes place by changing the degradation rate of CK by a constant c. (A) $c = 0.5$, (B) $c = 1$, (C-D) time evolution of meristem and OC radius after onset of over-expression.

CK loss of function. Fig. F shows time evolution of meristem and OC radius in the case of CK loss of function. As observed in experiments meristem and OC radius decrease.

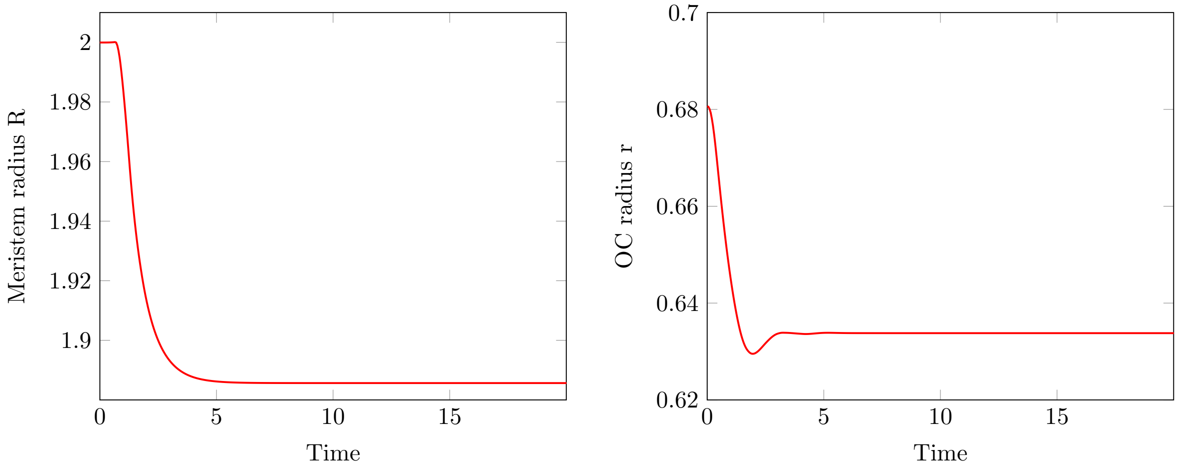

Figure F: **Meristem size in CK loss-of-function**. Time evolution of meristem and OC radius in absence of functional CK.

HEC over-expression in stem cells. As in experiments HEC over-expression in stem cells leads to an increase of the meristem radius and the OC. The OC radius approaches the radius of the the total meristem, Fig. G. Different levels of over-expression result in qualitatively similar dynamics. If we rerun the simulations omitting the direct effect of HEC on OC cell differentiation, the OC radius does not approach the meristem radius, see Fig. 8 (C-D) from main text. which is in contradiction to experimental observations [1].

To simulate latter scenario, in the ODE for r the HEC concentration was fixed to 0.3. This choice guaranties that the steady state for the modified model (without direct effect of HEC on OC cells) is identical to the steady state of the original model (with direct effect of HEC on OC cells).

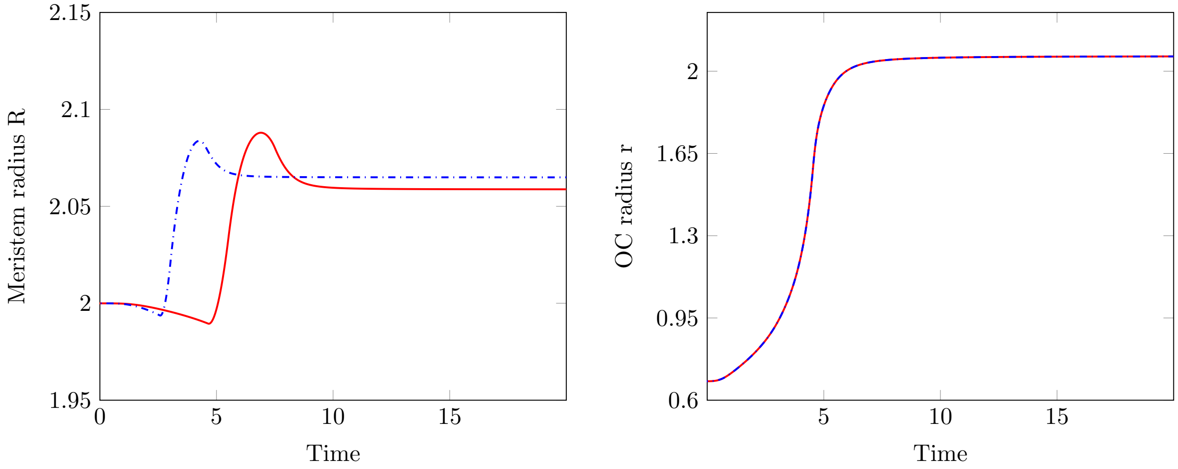

Figure G: **Meristem size in HEC over-expression.** Time evolution of meristem and OC radius during HEC over-expression in stem cells. The level of over-expression is determined by the positive constant c. Results are shown for $c = 0.05$ (solid red) and $c = 0.1$ (dashdotted blue).

HEC loss of function. HEC loss of function leads to a smaller radius of the OC and the total meristem, see Fig. H.

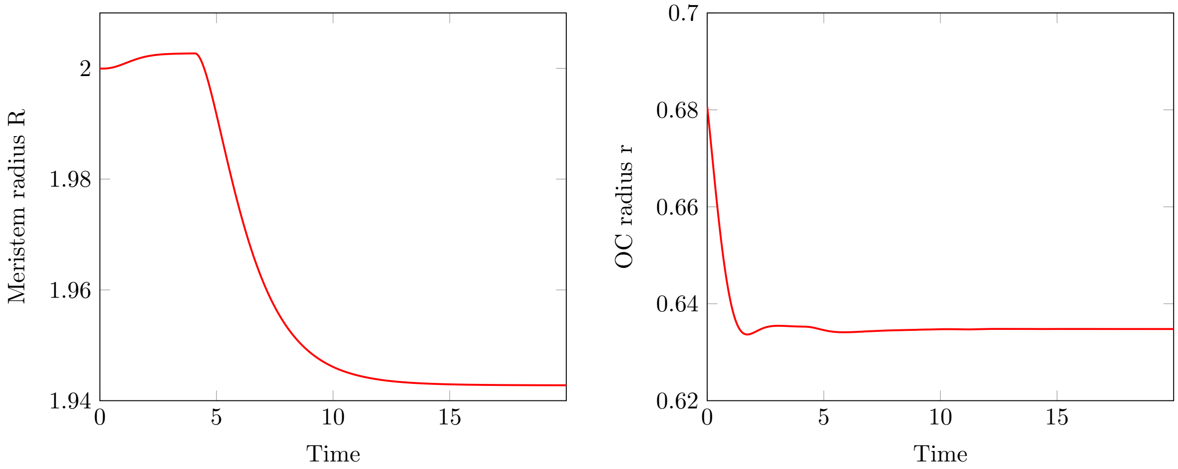

Figure H: **Meristem size in HEC loss of function.** Time evolution of meristem and OC radius in absence of functional HEC.

References

[1] C. Gaillochet, T. Stiehl, C. Wenzl, J.-J. Ripoll, L.J. Bailey-Steinitz, L. Li, A. Pfeiffer, A. Miotk, J.P. Hakenjos, J. Forner, M.F. Yanofsky, A. Marciniak-Czochra, and J.U. Lohmann. Control of plant cell fate transitions by transcriptional and hormonal signals. *Elife*, 6:e30135, 2017.

[2] R.K. Yadav, M. Tavakkoli, and G.V. Reddy. Wuschel mediates stem cell homeostasis by regulating stem cell number and patterns of cell division and differentiation of stem cell progenitors. *Development.*, 137(21):3581–9, 2010.
