# Supplementary material for: Mathematical modeling of plant cell fate transitions controlled by hormonal signals
Source: PLoS Comput Biol. 2020 Jul 20;16(7):e1007523. doi: 10.1371/journal.pcbi.1007523 (PMC7392350; doi:10.1371/journal.pcbi.1007523)
Supplement: S3 Text — (PDF) [file pcbi.1007523.s003.pdf]

# Supplementary Information S3 Text: Mathematical modeling of plant cell fate transitions controlled by hormonal signals

Filip Z. Klawe[1,⋆], Thomas Stiehl[1,2,3,⋆], Peter Bastian[2], Christophe Gaillochet[4], Jan U. Lohmann[5], and Anna Marciniak-Czochra[1,2,3]

[1] *Institute of Applied Mathematics, Heidelberg University, Heidelberg, Germany*
[2] *Interdisciplinary Center for Scientific Computing, Heidelberg University, Heidelberg, Germany*
[3] *Bioquant Center, Heidelberg University, Heidelberg, Germany*
[4] *VIB-UGent Center for Plant Systems Biology, Ghent University, Ghent, Belgium*
[5] *Department of Stem Cell Biology, Centre for Organismal Studies, Heidelberg University, Heidelberg, Germany*
⋆ *These authors contributed equally*

## Spatial dependence of proliferation rates.

The term $\frac{k_{21}}{1+k_{23}\int_{\Omega(t)} u_0 \,\mathrm{d}x}$ in equation (2) corresponds to the average proliferation rate in the SAM. To obtain the proliferation rate as a function of the radius we proceed as follows: We express the proliferation rate as a function of local WUS concentration, namely $p(x,t) = \frac{c}{(1+ku_0(x,t))}$. The parameter $k$ is chosen such that the ratio between proliferation rate in the central and proliferation rate in the peripheral zone agrees with measurements from [1]. This implies $k = 10$. The constant $c$ is chosen such that $\frac{1}{|\Omega(t)|}\int_{\Omega(t)} \frac{c}{(1+ku_0)}\,\mathrm{d}x = \frac{k_{21}}{1+k_{23}\int_{\Omega(t)} u_0 \,\mathrm{d}x}$. The obtained results are depicted in Figure A.

**Cell differentiation** We calibrated our parameters so that the ratio of the radius of the CZ and the radius of the SAM is equal to the experimentally measured value. In the simulations, the CZ is defined by high levels of CLV3. We define all cells with a CLV3 level not less than 10% of the CLV3 level in the centre of the steady state meristem as stem cells. This definition is in line with the observation that approximately 10% of meristem cells are located in the CZ.

The model considers cell differentiation happening at the outer boundary of the SAM where cells leave the SAM and enter into organs.

The outflux rate of cells from the SAM into organs is given by the term $pp\frac{1+R^2}{1+R_0^2}g(\int_{\Omega(t)} u_2 \,\mathrm{d}x)$ in equation (2).

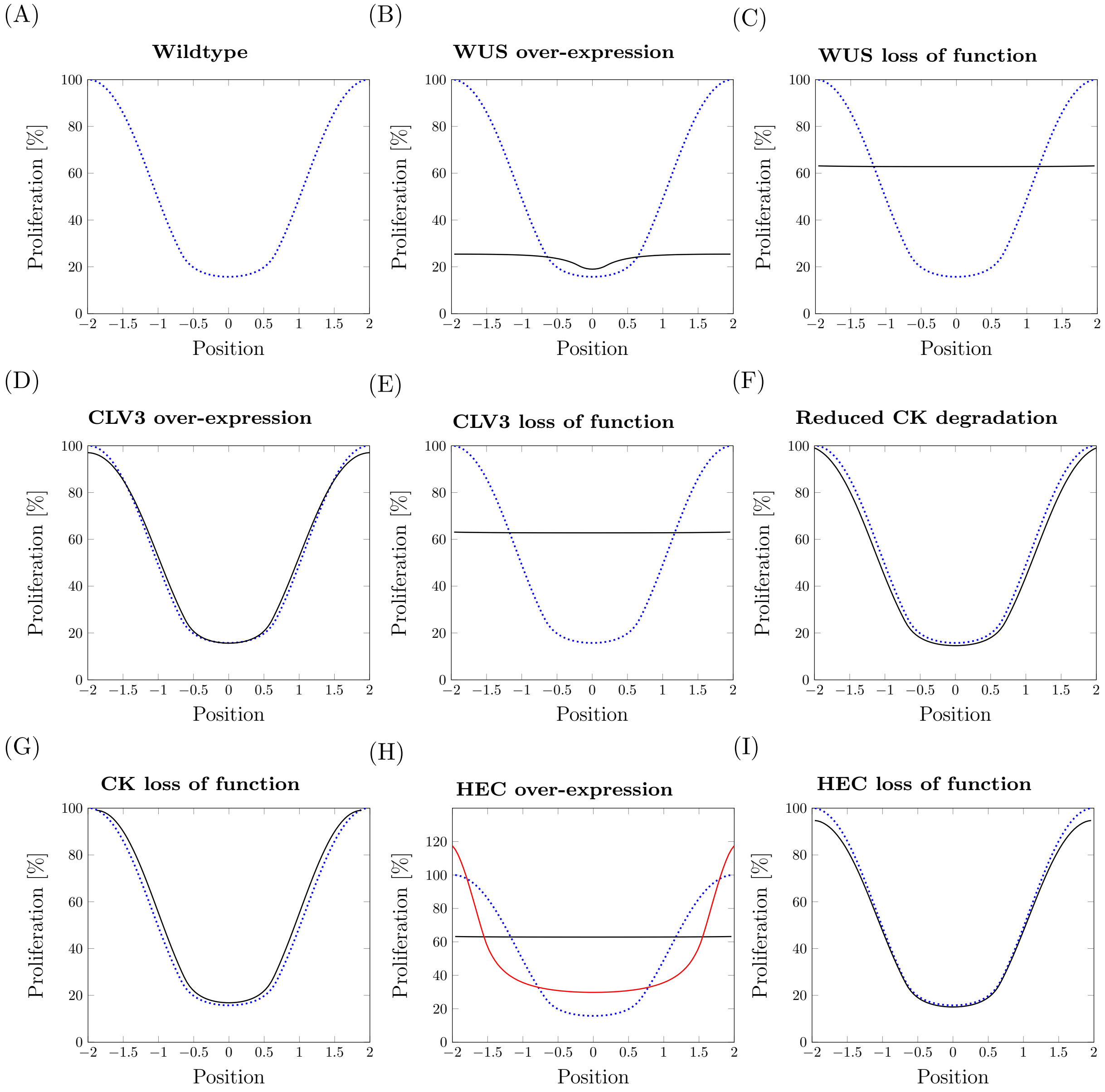


Figure A: **Spatial dependence of proliferation rates.** Proliferation rate are shown as a function of the radial distance from the SAM center. They have been normalized with respect to the maximum rate in the wild-type steady state. The blue dotted line shows the proliferation rate of the wild-type equilibrium. The black lines show the proliferation rates at the end of the respective numerical experiments. The proliferation rates correspond system states shown in Fig. 6 from main text. Dotted blue proliferation rate in steady state. (B) WUS over-expression $c = 2$ (C) WUS loss of function: red is with WUS=0 (D) CLV3 over-expression with $c = 2$ (E) CLV3 loss of function (F) Reduced CK degradation with $c = 1$ (G) CK loss of function (H) HEC over-expression with $c = 0.05$. The red line corresponds to the proliferation rate at the time when $r = 0.75R$. This is included because no measurements of proliferation rates at the end of the experiment are available for the HEC over-expression. (I) HEC loss of function.

# References

[1] G.V. Reddy, M.G. Heisler, D.W. Ehrhardt, and E.M. Meyerowitz. Real-time lineage analysis reveals oriented cell divisions associated with morphogenesis at the shoot apex of arabidopsis thaliana. *Development.*, 131(17):4225–4237, 2004.
