# Supplementary material for: Mathematical modeling of plant cell fate transitions controlled by hormonal signals
Source: PLoS Comput Biol. 2020 Jul 20;16(7):e1007523. doi: 10.1371/journal.pcbi.1007523 (PMC7392350; doi:10.1371/journal.pcbi.1007523)
Supplement: S4 Text — (PDF) [file pcbi.1007523.s004.pdf]

Supplementary Information S4 Text: Mathematical modeling of plant cell fate transitions controlled by hormonal signals

Filip Z. Klawe[1,⋆], Thomas Stiehl[1,2,3,⋆], Peter Bastian[2], Christophe Gaillochet[4], Jan U. Lohmann[5], and Anna Marciniak-Czochra[1,2,3]

[1] *Institute of Applied Mathematics, Heidelberg University, Heidelberg, Germany*
[2] *Interdisciplinary Center for Scientific Computing, Heidelberg University, Heidelberg, Germany*
[3] *Bioquant Center, Heidelberg University, Heidelberg, Germany*
[4] *VIB-UGent Center for Plant Systems Biology, Ghent University, Ghent, Belgium*
[5] *Department of Stem Cell Biology, Centre for Organismal Studies, Heidelberg University, Heidelberg, Germany*
⋆ *These authors contributed equally*

Description of the numerical scheme.

Continuously deforming domains. We model the continuously deforming domain $\Omega(t)$ for time $t > t_0$ as

$$\Omega(t) = \{x \,:\, x = X(\hat{x}, t) \text{ for some } \hat{x} \in \hat{\Omega}\}.$$

where $X(\hat{x}, t)$ is the push-forward map from the reference domain $\hat{\Omega} = \Omega(t_0)$ to time $t > t_0$. The push-forward map is given by the parametric ordinary differential equation

$$\frac{dX}{dt}(\hat{x}, t) = v(X(\hat{x}, t), t), \quad X(\hat{x}, t_0) = \hat{x}, \tag{1}$$

where $v : \Omega(t) \times t \to \mathbb{R}^d$ is a differentiable velocity field. Thus, the continuously deforming domain is given by the reference domain and the velocity field.

Strong form of the reaction-diffusion equation. The reaction-diffusion equation results from conservation of mass. Let $\omega(t) \subseteq \Omega(t)$ denote an arbitrary subdomain moving with the deformation (movement of points in ω is given by the push-forward map X). Conservation of a scalar quantity in $\omega(t)$ is stated by

$$\begin{aligned}\frac{d}{dt}\int_{\omega(t)} u(x,t)\,\mathrm{d}x &= -\int_{\partial\omega(t)} F(x,t)\cdot\nu(x,t)\,\mathrm{d}s - \int_{\omega(t)} f\big(u(x,t),x,t\big)\,\mathrm{d}x \\ &= -\int_{\omega(t)} \Big(\nabla\cdot F(x,t) + f\big(u(x,t),x,t\big)\Big)\,\mathrm{d}x,\end{aligned}$$

where $u(x,t)$ is the density of the quantity to be conserved, $f(u,x,t)$ is a source/sink/reaction term and $F(x,t)$ is the flux of the conserved quantity given by Fick's law

$$F(x,t) = -D(x,t)\nabla u(x,t)$$

since $\omega(t)$ moves with the flow. Inserting the flux law and employing Reynolds' Transport Theorem, we move the temporal derivative under the integral to obtain

$$\int_{\omega(t)} \Big(\frac{\partial u}{\partial t}(x,t) + \nabla\cdot(u(x,t)v(x,t)) - \nabla\cdot D(x,t)\nabla u(x,t) + f\big(u(x,t),x,t\big)\Big)\,\mathrm{d}x = 0.$$

Since $\omega(t)$ was arbitrary the integrand must be zero pointwise (fundamental theorem of calculus) and we obtain the strong form of the reaction-diffusion equation on deforming domains:

$$\frac{\partial u}{\partial t}(x,t) + \nabla\cdot(u(x,t)v(x,t)) - D(x,t)\nabla u(x,t)) + f(u(x,t),x,t) = 0 \qquad x\in\Omega(t). \tag{2}$$

This equation is supplemented with initial conditions and boundary conditions which we assume to be of Neumann type for simplicity of notation:

$$u(x,t_0) = u_0(x)\ \ (x\in\Omega(t_0)), \qquad \frac{\partial u(x,t)}{\partial\vec{n}} = 0\ \ (x\in\partial\Omega(t)).$$

Weak form of the reaction-diffusion equation. We first need to derive some auxiliary results. The inverse of the push-forward map is denoted by $X^{-1}(x,t)$. It maps point x at time t to a point in the reference domain. We assume the following identities hold (invertibility of push-forward map):

$$X^{-1}(X(\hat{x},t),t) = \hat{x} \quad\Leftrightarrow\quad X(X^{-1}(x,t),t) = x \quad (t\in[t_0,t_0+T_I]). \tag{3}$$

Differentiating the identity on the right by t and using the chain rule we obtain

$$\frac{\partial X}{\partial t}(X^{-1}(x,t),t) + \hat{\nabla}X(X^{-1}(x,t),t)\frac{\partial X^{-1}}{\partial t}(x,t) = 0$$

where $\hat{\nabla}$ denotes the jacobian differentiating with respect to coordinates in the reference domain. Rearranging and using the definition of X from (S1) we obtain

$$\frac{\partial X^{-1}}{\partial t}(x,t) = -\Big(\hat{\nabla}X(X^{-1}(x,t),t)\Big)^{-1} v(x,t) = -\nabla X^{-1}(x,t)v(x,t) \tag{4}$$

where used $\nabla X^{-1}(X(\hat{x},t),t) = \Big(\hat{\nabla}X(\hat{x},t)\Big)^{-1}$ in the last step which is obtained from differentiating the left identity in (S3) w.r.t. to all coordinates.

Next, consider a function $w(x,t)$ on the continuously deforming domain by linking it to a function $\hat{w}(\hat{x},t)$ defined on the reference domain and any time, i.e.:

$$w(x,t) = \hat{w}(X^{-1}(x,t),t) \quad\Leftrightarrow\quad w(X(\hat{x},t),t) = \hat{w}(\hat{x},t). \tag{5}$$

Applying the chain rule to the second identity we obtain the well-known transformation formula for gradients

$$\nabla w(X(\hat{x},t),t) = \left(\hat{\nabla} X(\hat{x},t)\right)^{-T} \hat{\nabla}\hat{w}(\hat{x},t) \tag{6}$$

which allows to compute the gradient of a w on the deformed domain from the gradient of $\hat{w}$ and the jacobian of the push-forward map at time t.

Applying the chain rule to the time derivative of the first identity in (S5) and using the identities derived above yields the following important formula:

$$\begin{aligned}
\frac{\partial w}{\partial t}(x,t) &= \frac{d\hat{w}}{dt}(X^{-1}(x,t),t) \\
&= \frac{\partial \hat{w}}{\partial t}(X^{-1}(x,t),t) + \hat{\nabla}\hat{w}(X^{-1}(x,t),t)\cdot\frac{\partial X^{-1}}{\partial t}(x,t) \\
&= \frac{\partial \hat{w}}{\partial t}(X^{-1}(x,t),t) - \hat{\nabla}\hat{w}(X^{-1}(x,t),t)\cdot\left[\left(\hat{\nabla}X(X^{-1}(x,t),t)\right)^{-1} v(x,t)\right] \\
&= \frac{\partial \hat{w}}{\partial t}(X^{-1}(x,t),t) - \left[\left(\hat{\nabla}X(X^{-1}(x,t),t)\right)^{-T} \hat{\nabla}\hat{w}(X^{-1}(x,t),t)\right]\cdot v(x,t) \\
&= \frac{\partial \hat{w}}{\partial t}(X^{-1}(x,t),t) - \nabla w(x,t)\cdot v(x,t).
\end{aligned} \tag{7}$$

Using this result we derive a corollary of the Reynolds transport theorem. Formally applying the Reynolds transport theorem to the product $u(x,t)w(x,t)$, where the dependence of w on time is assumed to be only due to domain deformation, i.e. $w(x,t) = \hat{w}(X^{-1}(x,t))$, yields

$$\begin{aligned}
&\frac{d}{dt}\int_{\Omega(t)} u(x,t)w(x,t)\,\mathrm{d}x \\
&\quad= \int_{\Omega(t)} \Big(\frac{\partial(uw)}{\partial t}(x,t) + \nabla\cdot\big(u(x,t)w(x,t)v(x,t)\big)\Big)\,\mathrm{d}x \\
&\quad= \int_{\Omega(t)} \Big(\frac{\partial u}{\partial t}(x,t)w(x,t) + u(x,t)\frac{\partial w}{\partial t}(x,t) + \nabla\cdot\big(u(x,t)w(x,t)v(x,t)\big)\Big)\,\mathrm{d}x \\
&\quad= \int_{\Omega(t)} \Big(\frac{\partial u}{\partial t}(x,t)w(x,t) - u(x,t)\nabla w(x,t)\cdot v(x,t) + \nabla\cdot\big(u(x,t)w(x,t)v(x,t)\big)\Big)\,\mathrm{d}x \\
&\quad= \int_{\Omega(t)} \Big(\frac{\partial u}{\partial t}(x,t)w(x,t) + \nabla\cdot\big(u(x,t)v(x,t)\big)w(x,t)\Big)\,\mathrm{d}x.
\end{aligned}$$

We are now in a position to derive the weak formulation of (S2). Multiplying by a test function $w(\cdot,t) \in H^1(\Omega(t))$ (depending on time only via the deformation of the domain), integrating by parts and using the

corollary of Reynolds' transport theorem yields:

$$\begin{aligned}
&\int_{\Omega(t)} \left[\frac{\partial u}{\partial t}(x,t) + \nabla\cdot\Big(u(x,t)v(x,t) - D(x,t)\nabla u(x,t)\Big) + f\big(u(x,t),x,t\big)\right] w(x,t)\,\mathrm{d}x \\
&\quad = \int_{\Omega(t)} \Big(\frac{\partial u}{\partial t}(x,t)w(x,t) + \nabla\cdot\big(u(x,t)v(x,t)\big)w(x,t) \\
&\qquad\qquad + (D(x,t)\nabla u(x,t))\cdot\nabla w(x,t) + f\big(u(x,t),x,t\big)w(x,t)\Big)\,\mathrm{d}x \\
&\quad = \frac{d}{dt}\int_{\Omega(t)} u(x,t)w(x,t)\,\mathrm{d}x \\
&\qquad\qquad + \int_{\Omega(t)} \Big(\big(D(x,t)\nabla u(x,t)\big)\cdot\nabla w(x,t) + f\big(u(x,t),x,t\big)w(x,t)\Big)\,\mathrm{d}x.
\end{aligned}$$

Setting $U(t) = H^1(\Omega(t))$ and $V(t) = H^1(\Omega(t))$, the weak formulation seeks $u \in L_2([t_0, t_0+T]; U(t))$ such that for all $t \in (t_0, t_0+T]$

$$\begin{aligned}
&\frac{d}{dt}\int_{\Omega(t)} u(x,t)w(x,t)\,\mathrm{d}x + \int_{\Omega(t)} \big(D(x,t)\nabla u(x,t)\big)\cdot\nabla w(x,t)\,\mathrm{d}x \\
&\qquad + \int_{\Omega(t)} f\big(u(x,t),x,t\big)w(x,t)\,\mathrm{d}x = 0 \qquad \forall w(\cdot,t)\in V(t).
\end{aligned} \tag{8}$$

Observe that we chose to keep the temporal derivative outside of the integral.

Finite element formulation. The FEM approximates the solution in a finite-dimensional subspace defined on a mesh. Here we assume that a mesh is obtained for the reference domain $\Omega(t_0)$ which is then continuously deforming with the domain. The time-dependent mesh is denoted by its elements

$$\mathcal{T}_h(t) = \{T_0(t), \dots, T_{m-1}(t)\}$$

and the nodal positions

$$\mathcal{N}_h(t) = \{N_0(t), \dots, N_{n-1}(t)\}.$$

The connectivity of elements to nodes is given by the local to global map

$$g_T : \{0, \dots, \hat{n}-1\} \to \{0, \dots, n-1\}$$

mapping the $\hat{n}$ node numbers of the reference element $\hat{T}$ to global node numbers. The local to global numbering does not change throughout the computation and it is assumed that the quality of the mesh does not deteriorate with time (a reasonable assumption for the problem here).

Setting up the finite element problem is done by a pull-back to the reference element $\hat{T}$ as usual. To that end define the map

$$\mu_T(\hat{x}, t) = \sum_{l=0}^{\hat{n}-1} \hat{\psi}_l(\hat{x}) N_{g_T(l)}(t) \tag{9}$$

mapping a point $\hat{x}$ in the reference element $\hat{T}$ to the mesh element T at time t. Here, $\hat{\psi}_i(\hat{x})$ are the (multi-)linear Lagrange shape functions on the reference element. Consequently, the approximation $\Omega_h(t)$ of the

domain $\Omega(t)$ is polygonal (this could be easily extended to higher order elements but we refrain from doing so in order to keep the notation simple).

Now we are in a position to define the lowest order conforming finite element space on the continuously deforming mesh via the element transformation map:

$$V_h(t) = \{w \in C^0(\Omega(t)) \,:\, w|_T = p \circ \mu_T^{-1}, T \in \mathcal{T}_h(t), p \in \mathbb{Q}_1\} \tag{10}$$

with $\mathbb{Q}_1$ the multi-linear polynomials in dimension d (simplicial grids and piecewise linear can be used as well). For the test and ansatz functions we employ the subspace and affine subspace: $W_h(t) = V_h(t)$ and $U_h(t) = V_h(t)$. The time-continuous finite element problem now consists in finding $u(x,t) \in L_2([t_0, t_0 + T]; U_h(t))$ such that for all $t \in (t_0, t_0 + T]$

$$\begin{aligned} \frac{d}{dt}\int_{\Omega_h(t)} u_h(x,t) w(x,t)\,\mathrm{d}x + \int_{\Omega_h(t)} \big(D(x,t)\nabla u_h(x,t)\big)\cdot\nabla w(x,t)\,\mathrm{d}x \\ + \int_{\Omega_h(t)} f\big(u_h(x,t), x, t\big) w(x,t)\,\mathrm{d}x = 0 \qquad \forall w(\cdot,t) \in W_h(t). \end{aligned} \tag{11}$$

Choosing a basis representation

$$V_h(t) = \mathrm{span}\{\varphi_i(\cdot,t) \,:\, 0 \le i < n\}$$

and inserting the ansatz

$$u_h(x,t) = \sum_{j=0}^{n-1} z_j(t)\varphi_j(x,t).$$

in (S11) results in a system of ordinary differential equations (ODE) for the unknown coefficients $z_j(t)$.

For discretization in time introduce the possibly non-equidistant time steps

$$t_0 = t^0 < t^1 < \ldots < t^q = t_0 + T, \qquad \tau^k = t^{k+1} - t^k. \tag{12}$$

Denoting position of node i at time t^k by N_i^k nodal positions are interpolated in time

$$N_i(t) = \sum_{k=0}^{q} \xi_k(t) N_i^k$$

where the $\xi_k(t)$ are the one-dimensional, piecewise linear basis functions on the subdivision (S12).

Given discrete nodal positions N_i^k at t^k and N_i^{k+1} at t^{k+1}, nodal positions are interpolated linearly

$$N_i(t) = \frac{t^{k+1} - t}{\tau^k} N_i^k + \frac{t - t^k}{\tau^k} N_i^{k+1}$$

and we solve the system of ODEs using a second-order accurate diagonally implicit Runge-Kutta (DIRK) method. The required integrals are computed efficiently with the pull-back to the reference element given in (S9).

Operator splitting scheme for coupled problem. Above nodal positions N_i^k were given. We now treat the case where the nodal positions depend on the solution of the PDE. In the problem considered in

this paper the domain is a circle with time-dependend radius $r(t)$ centered at the origin. The change in radius is given a relation of the form

$$\frac{dR}{dt}(t) = \int_{\Omega(t)} G(u(x,t), x, t)\, dx \tag{13}$$

with some function $G(u, x, t)$.

Coupling of the PDE and the domain growth is rather weak and we use first-order operator splitting scheme in time and explicit Euler for solving (S13):

1. Generate mesh $\mathcal{T}_h(t_0)$ with nodal positions N_i^0 for $\Omega(t_0)$ and initialize $u_h(\cdot, t_0)$ from initial value u_0. Set $k = 0$ and choose a fixed time step $\tau^k = \tau$.

2. Given nodal positions N_i^k and $u_h(\cdot, t^k)$, compute a new radius via

$$R(t^{k+1}) = R(t^k) + \tau^k \int_{\Omega(t)} G(u_h(x, t^k), x, t^k)\, dx$$

as well as new nodal positions

$$N_i^{k+1} = N_i^k + \tau^k \frac{N_i^k}{r(t^k)} \frac{r(t^{k+1}) - r(t^k)}{\tau^k} = N_i^k \left(1 + \frac{r(t^{k+1}) - r(t^k)}{r(t^k)}\right)$$

using the explicit Euler scheme.

3. Solve the PDE problem in $(t^k, t^{k+1}]$ and go to 2.
