# Supplementary material for: Mathematical modeling of plant cell fate transitions controlled by hormonal signals
Source: PLoS Comput Biol. 2020 Jul 20;16(7):e1007523. doi: 10.1371/journal.pcbi.1007523 (PMC7392350; doi:10.1371/journal.pcbi.1007523)
Supplement: S5 Text — (PDF) [file pcbi.1007523.s005.pdf]

# Supplementary Information S5 Text: Mathematical modeling of plant cell fate transitions controlled by hormonal signals

Filip Z. Klawe[1,⋆], Thomas Stiehl[1,2,3,⋆], Peter Bastian[2], Christophe Gaillochet[4], Jan U. Lohmann[5], and Anna Marciniak-Czochra[1,2,3]

[1] *Institute of Applied Mathematics, Heidelberg University, Heidelberg, Germany*
[2] *Interdisciplinary Center for Scientific Computing, Heidelberg University, Heidelberg, Germany*
[3] *Bioquant Center, Heidelberg University, Heidelberg, Germany*
[4] *VIB-UGent Center for Plant Systems Biology, Ghent University, Ghent, Belgium*
[5] *Department of Stem Cell Biology, Centre for Organismal Studies, Heidelberg University, Heidelberg, Germany*
[⋆] *These authors contributed equally*

## Sensitivity.

To study the sensitivity of the model we performed numerical experiments with perturbed parameters. In each simulation we perturbed all of the parameters by a random value from the interval $[-p, p]$, where $p$ was set to 2.5% and 5% of the respective parameter value. After 100 and 103 simulations (for $p$ equal to 2.5% and 5%, respectively) we calculated the 95% confidence intervals (the 95% quantiles of the normal distribution with the mean value and standard deviation obtained from the sample). The obtained results are presented in Table A. Obtained ratios between radii were normalized by division with the mean value of the sample. In Fig. A we present confidence intervals for $R$, $r$ and the ratio between $r$ and $R$. All quantities are divided by the mean values of the sample.

Sensitivity analysis suggests that the outer radius is more robust to perturbations of the parameters compared to the inner radius. This implies that the regulations considered in the model tend to conserve the total number of cells in the outer meristem layers. However, the size of the organizing center and hence the proportion of stem cells among all cells may vary in response to perturbations of model parameters.

All simulations were run on the mesh $N = 50$ (90404 degrees of freedom) with time step $dt = 0.05$. The variation in the ratio $r/R$ is of the same order of magnitude as the perturbation of the parameters. This implies that the model is robust with respect to perturbations of parameter values.

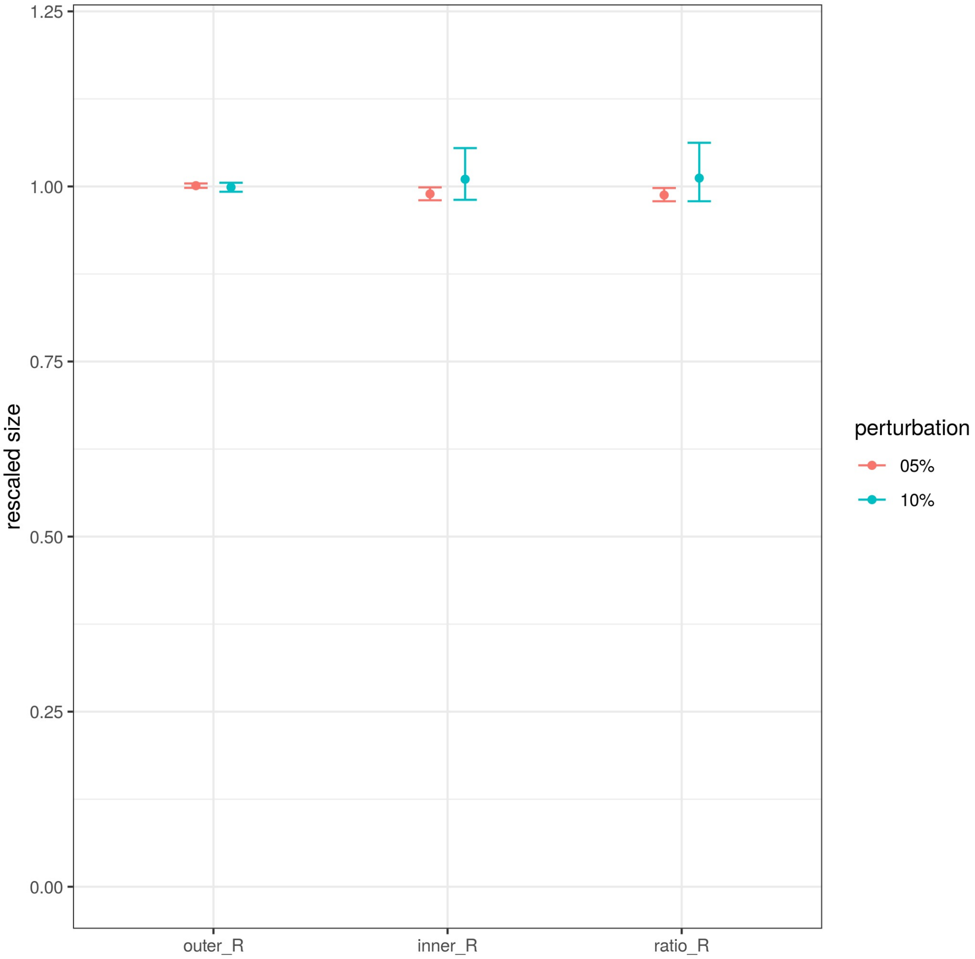


Figure A: **Sensitivity analysis.** 95% confidence interval for perturbation $\pm 2.5\%$ (red line 05%) and $\pm 5\%$ (blue line 10%). All values are presented on rescaled axes.

| perturbation | $p = 2.5\%$ | $p = 5\%$ |
|---|---|---|
| number of experiments | 100 | 103 |
| 95% confidence interval | $[0.98997, 1.01003]$ | $[0.96031, 1.03969]$ |
| 95% confidence interval -1 | $[-1.0025\%, 1.0025\%]$ | $[-3.9695\%, 3.9695\%]$ |

Table A: **Changes of the ratio $r/R$ for randomly perturbed model parameters.** Changes of the ratio $r/R$ if model parameters are randomly perturbed with values from the interval $[-p, p]$. For $p \in \{2.5\%,\ 5\%\}$ at least 100 numerical experiments were simulated. The numerical value $r/R$ assumes in the system with unperturbed parameters simulated on the same mesh with the same time step corresponds to 100%. It equals 0.3403.
